# Educational Intervention on Environmentally Responsible Inhaler Prescribing Among French General Practitioners: Pilot Pre-Post Study

**DOI:** 10.2196/89593

**Published:** 2026-06-11

**Authors:** Camille Lapeyre, Aurélie Urena-Dores, Arnaud Bourdin, Jean-Baptiste Tostain, François Carbonnel

**Affiliations:** 1 Department of General Practice, Faculty of Medicine of Montpellier-Nîmes University of Montpellier Montpellier, Occitanie France; 2 Department of Pulmonology, Faculty of Medicine of Montpellier-Nîmes University of Montpellier Montpellier, Occitanie France

**Keywords:** hydrofluoroalkane, greenhouse gas, metered-dose inhalers, dry powder inhalers, asthma, chronic obstructive pulmonary disease, pilot study, primary health care, general practice

## Abstract

**Background:**

Climate change is expected to cause more than 250,000 deaths annually by 2050 and could increase the prevalence of asthma and chronic obstructive pulmonary disease (COPD) by up to 30%. Pressurized metered-dose inhalers (pMDIs), primarily delivering short-acting beta-2 agonists, generate 15 to 30 times more greenhouse gas emissions than dry powder or soft mist inhalers. In France, short-acting beta-2 agonist pMDIs account for 95% of reliever therapy prescriptions, despite their limited effectiveness in controlling disease symptoms.

**Objective:**

This study aimed to evaluate the preliminary educational impact of a single educational session on French general practitioners’ awareness and intended prescribing of lower-carbon inhaler alternatives.

**Methods:**

We conducted a multicenter, single-group pre-post pilot study among 34 general practitioners from 10 multiprofessional health centers in Eastern Occitanie, France, between March and October 2023. Participants were recruited through convenience sampling. The intervention consisted of a one-time 25-minute face-to-face educational session on environmentally responsible inhaler prescribing, aligned with Global Initiative for Asthma (GINA) and Global Initiative for Chronic Obstructive Lung Disease guidelines. Data were collected using self-administered online questionnaires before the intervention and approximately 3 months later. The questionnaires included 2 clinical vignettes, one on asthma and one on COPD, with 3 prescribing questions each. Responses were categorized according to whether they included a pMDI. Changes in responses between baseline and follow-up were analyzed using the Fisher exact test or chi-square test, as appropriate.

**Results:**

A total of 34 participants completed the baseline questionnaire. Responses including a pMDI decreased from 70.6% (48/68) to 4% (3/68) for reliever therapy (*P*<.001) and from 21.3% (29/136) to 4.4% (6/136) for maintenance therapy (*P*=.003). In asthma scenarios, adherence to GINA recommendations improved, with increased responses including inhaled corticosteroid-formoterol for reliever therapy (6%, 2/34 to 38%, 13/34; *P*=.001) and maintenance therapy (35%, 24/68 to 56%, 38/68; *P*=.02). No significant improvements were observed for COPD-related prescribing scenarios. The proportion of participants reporting environmental impact as a factor influencing inhaler choice increased from 3% (1/34) to 51% (18/34). Satisfaction was high, with 93% of participants reporting being very satisfied with the intervention.

**Conclusions:**

This pilot study suggests that a brief educational intervention may improve general practitioners’ knowledge and intended prescribing of lower-carbon inhaler alternatives, particularly in asthma scenarios. However, the outcomes were based on theoretical clinical vignettes rather than real-world prescribing data, and the study was not designed to assess the safety or clinical effectiveness of changing inhaler prescriptions. Future studies should evaluate sustained changes in real-world prescribing while ensuring individualized, clinically appropriate, and safe inhaler choices.

## Introduction

Climate change is projected to become the leading threat to human health over the coming decades according to the World Health Organization [1]. It may cause more than 250,000 additional deaths annually between 2030 and 2050, particularly through respiratory, cardiovascular, and infectious diseases. Chronic respiratory diseases, including asthma and chronic obstructive pulmonary disease (COPD), are expected to increase by 30% by 2050 [2,3]. The health care sector also contributes to greenhouse gas emissions. In France, it accounts for 8% of national greenhouse gas emissions, with medication use representing the largest contributor, linked to the sale of approximately 3.1 billion boxes annually and accounting for 30% of the sector’s carbon footprint [4-6].

**Figure 1:**
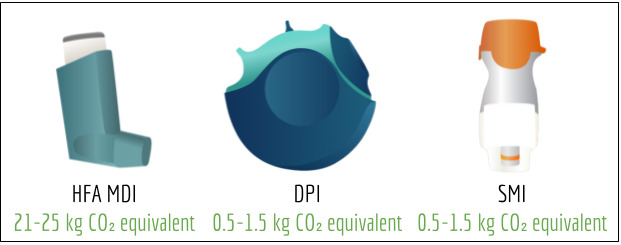
Main types of inhalers and their estimated carbon footprints. The graphical illustration was author-generated with assistance from generative artificial intelligence tools. The CO2-equivalent values were derived from published scientific references, including Woodcock et al [11] and Tirumalasetty et al [15]. DPI: dry powder inhaler; HFA MDI: hydrofluoroalkane metered-dose inhaler; SMI: soft mist inhaler.

Asthma and COPD affect approximately 8 million people in France [7,8]. Inhalers are the cornerstone of treatment for these conditions, with 41 million devices sold annually [9,10]. Three main types of inhalers are currently available: pressurized metered-dose inhalers (pMDIs), dry powder inhalers (DPIs), and soft mist inhalers (SMIs). Unlike DPIs and SMIs, pMDIs contain hydrofluoroalkanes (HFA; notably HFA-134a and HFA-227ea), which have a global warming potential 1300 to 3350 times higher than that of carbon dioxide [11]. As a result, they emit 15 to 30 times more greenhouse gases than DPI or SMI alternatives, despite comparable clinical efficacy in many situations [12-14] (Figure 1 [11,15]). The carbon footprint of a single pMDI is estimated to be equivalent to that of a 300 km journey by car [16].

pMDIs remain the most frequently prescribed option, accounting for approximately 45% of inhaler prescriptions in both France and Europe [17,18]. They are particularly prescribed in acute care, accounting for 95% of reliever prescriptions, primarily with short-acting beta-2 agonists (SABA). Recent Global Initiative for Asthma (GINA) recommendations encourage reducing SABA overuse and promoting anti-inflammatory reliever strategies [19]. However, in maintenance therapy, the safety of switching a stable patient from a pMDI to a DPI remains widely debated. Although previous studies have shown overall safety in retrospective analyses [20], several recent publications have raised concerns about such switches in older patients, noting a 10% increase in respiratory-related hospitalizations [21]. Transitioning from SABA pMDIs to the new GINA-recommended anti-inflammatory reliever and maintenance and reliever therapy could reduce CO_2_ emissions by 42% [22].

Prescribing patterns vary substantially across countries. For example, in Sweden, 90% of inhaler prescriptions are for DPIs, compared with 75% for pMDIs in the United Kingdom [20]. Several countries have implemented initiatives promoting environmentally sustainable inhaler prescribing, including the Greener Practice initiative in the United Kingdom and the Cascades program in Canada [16,23,24]. GINA has also recently integrated environmental considerations into its latest guidelines. To date, no comparable national initiative has emerged in France [19].

General practitioners (GPs) are responsible for 77.6% of inhaler prescriptions and therefore play a central role in promoting sustainable respiratory care [20,25]. Previous studies suggest that educational interventions may improve physicians’ awareness of environmental health issues and influence prescribing intentions [26]. To our knowledge, no French study has evaluated an educational intervention targeting environmentally responsible inhaler prescribing in primary care.

The primary objective of this pilot study was to evaluate the preliminary impact of a brief, face-to-face educational session on GPs’ knowledge and intended prescribing of lower-carbon inhaler alternatives in theoretical clinical scenarios. Secondary objectives included participant satisfaction and perceived usefulness of the intervention.

## Methods

### Trial Design

We conducted a pilot study using a single-group, pre-post, noncontrolled, multicenter formative design. It consisted of a single 25-minute face-to-face educational session on environmentally responsible inhaler prescribing. The study was reported in accordance with the CONSORT (Consolidated Standards of Reporting Trials) 2010 extension for pilot and feasibility trials, excluding items related to randomization, which were considered not applicable in this context (Multimedia Appendix 1) [27].

To guide the design and evaluation of the educational intervention, we used the Kirkpatrick four-level training evaluation model, a well-established framework for assessing educational programs [28]. The model assesses 4 key dimensions of training effectiveness: reaction, defined as the extent to which participants perceive the training as engaging, relevant, and valuable to their professional practice; learning, referring to the acquisition of the intended knowledge, skills, and confidence; behavior, which reflects the degree to which participants integrate and apply the acquired competencies in clinical practice; and results, corresponding to the achievement of targeted clinical or organizational outcomes attributable to the training and associated support measures.

### Participants

The study took place in the Eastern Occitanie region of France. Recruitment occurred between March 2023 and September 2023, and follow-up concluded in March 2024. Eligible participants were GPs and GP residents working in multiprofessional health centers (MPHCs). Participants were recruited using convenience sampling. The principal investigator (CL) contacted the different MPHCs. Contact details were provided by the Regional Health Agency. Of the 36 centers identified by the Regional Health Agency, 17 (47.2%) were excluded because they were located more than a 90-minute drive away. The remaining centers were contacted by telephone or email. If no response was obtained, the center was contacted again 1 week later. In the absence of a second response, a third contact attempt was made 1 week later.

During each telephone contact, the intervention was briefly presented as an educational initiative conducted as part of a doctoral thesis, aiming to raise physicians’ awareness of prescribing low-carbon inhalers for asthma and COPD.

The discussion could take place either with the secretary or directly with the center coordinator. If the center coordinator was interested in participating, they expressed their interest by phone or email. The coordinator was then responsible for informing the other physicians within the center about the study. Once physicians from the center had expressed interest in participating, the coordinator contacted the investigator to schedule a date for the intervention. If no follow-up was received within 2 weeks, the investigator contacted the coordinator again proactively. Participation was voluntary.

### Interventions

The educational intervention consisted of a single 25-minute face-to-face educational session on environmentally responsible inhaler prescribing, aligned with GINA and Global Initiative for Chronic Obstructive Lung Disease (GOLD) guidelines. It was delivered on-site at each participating MPHC, followed by an optional discussion period. The content is summarized in Textbox 1 and detailed in Multimedia Appendix 2.

**Structure of educational intervention. The educational intervention was delivered orally in participating medical centers and was supported by a PowerPoint presentation composed of illustrative slides.**

**Introduction**

- Greenhouse gases, climate change, and health care–related emissions (5 min)

**Inhalers**

- Inhaler types, comparative clinical efficacy, and carbon footprint (5-10 min)
- European and French prescribing trends (5 min)

**Clinical Cases**

- Two clinical vignettes focusing on asthma and chronic obstructive pulmonary disease were used to support the review of current Global Initiative for Asthma and Global Initiative for Chronic Obstructive Lung Disease recommendations (10 min)

**Discussion**

- Optional question-and-answer session and peer discussion (5-30 min)

All sessions were conducted by the same presenter (CL) to ensure consistency. The first presentation was delivered and validated by FC, head of the Department of General Practice at the University of Montpellier. A second version was then developed under the supervision of both AB, head of Pulmonology at Montpellier University Hospital, and FC. The new version incorporated the latest GINA and GOLD recommendations.

The presenter (CL) completed a day of therapeutic education consultations in the pulmonology department to familiarize himself with the different inhalers. The session included a narrated slide presentation, visual prescribing aids derived from current GINA and GOLD guidelines, as well as a video hosted on YouTube to provide additional reinforcement (Multimedia Appendix 2).

### Data Collection

A literature review was conducted before implementing the study to identify validated instruments appropriate for assessing ecological awareness. As none were fully appropriate for our objectives and target population, 2 custom questionnaires were developed by the research team, based on existing tools and international recommendations [29,30].

Data were collected through 2 self-administered online questionnaires using LimeSurvey (version 6.4.12; LimeSurvey GmbH). Responses were anonymized using a secure correspondence table. The preintervention questionnaire was completed before the educational intervention. Participants who had not completed the questionnaire before the day of the intervention were asked to complete it prior to the start of the session. The questionnaire link was sent after the intervention date had been scheduled.

The postintervention questionnaire was completed approximately 3 months later (Multimedia Appendix 3). Participants received individual follow-up reminders by phone or email every 2 weeks until the questionnaire was completed.

To illustrate the new recommendations and therapeutic options, we created 2 clinical vignettes, one focusing on asthma and the other on COPD. Each vignette contained 3 prescribing questions, for a total of 6 questions overall.

The preintervention questionnaire comprised 20 items organized into 3 sections (Multimedia Appendix 3). The first section collected demographic characteristics, including age, gender, and years of practice. The second section assessed participants’ knowledge regarding inhaler types, greenhouse gas emissions, and clinical effectiveness. The third section consisted of clinical scenarios based on 2 case vignettes involving asthma and COPD, each including one reliever treatment question and 2 maintenance treatment questions. Only mild chronic respiratory diseases were considered, as severe cases generally require specialist management, and all hypothetical patients were treatment-naive.

The postintervention questionnaire comprised 15 items evaluating participants’ satisfaction with and perceived utility of the intervention using 4-point Likert scales, as well as repeated knowledge questions designed to assess changes in knowledge acquisition and intended prescribing behavior, in accordance with levels 2 and 3 of the Kirkpatrick model.

In addition to closed-ended questions, the questionnaire included an open-ended item and a free-text section, enabling participants to provide additional comments and qualitative insights.

### Outcomes

The primary outcome was assessed using a questionnaire based on 2 clinical cases. Physicians were asked to indicate which inhaler they would prescribe for each scenario. Responses were subsequently categorized according to whether or not they included a pMDI. In total, the questionnaire comprised 6 prescribing questions. The key secondary outcome was to evaluate improvements in adherence to the GINA asthma guidelines.

Additional secondary outcomes included adherence to GOLD recommendations for COPD, changes in intended prescribing behavior and associated influencing factors, knowledge acquisition, and participants’ satisfaction with and perceived usefulness of the training intervention.

### Sample Size

On the basis of data indicating that 45% of inhaler prescriptions in France involve pMDIs [20], and assuming an absolute 20-percentage-point reduction after the intervention [31-33], we estimated that a minimum sample size of 29 participants would provide sufficient power to detect a preliminary educational effect using a 1-sided alpha level of 5% and 80% power.

The sample size calculation was performed prior to the study and guided the recruitment process.

### Statistical Methods

Categorical variables were compared using the Fisher exact test when n<5 and chi-square tests when n≥5. Analyses were performed using BiostatGV, an open-access web-based statistical tool.

Missing data were handled conservatively by assuming no change in participant responses, using an approach similar to baseline observation carried forward. A sensitivity analysis was subsequently performed using a complete-case analysis that included only participants who completed both the baseline and follow-up questionnaires.

Because multiple prescribing responses were provided by the same participants across different clinical scenarios, observations were not fully independent. Therefore, inferential statistics should be interpreted cautiously and primarily as exploratory.

Secondary analyses were performed to explore potential heterogeneity in the effect of the educational intervention according to participants’ characteristics. Subgroups were defined a priori based on age, sex, years of practice, practice location, and the presence of a pulmonologist within the MPHC.

### Ethical Considerations

According to the French Public Health Code (Article L1121-1), submission to an ethics committee was not required, as the study fell outside the scope of research involving human participants because no patient data were collected and no clinical intervention was performed.

All participants received information about the study and provided informed consent prior to participation. Data were anonymized and securely stored on a password-protected external drive. No financial compensation was provided for participation.

## Results

### Recruitment and Participant Flow

Recruitment took place from March 1 to September 19, 2023, when the targeted sample size was reached.

The median time between the educational intervention and completion of the posttest questionnaire was 4 (IQR 3.5-4.5) months. The mean duration of the educational session, including discussion, was 40 (SD 20.3) minutes.

**Figure 2:**
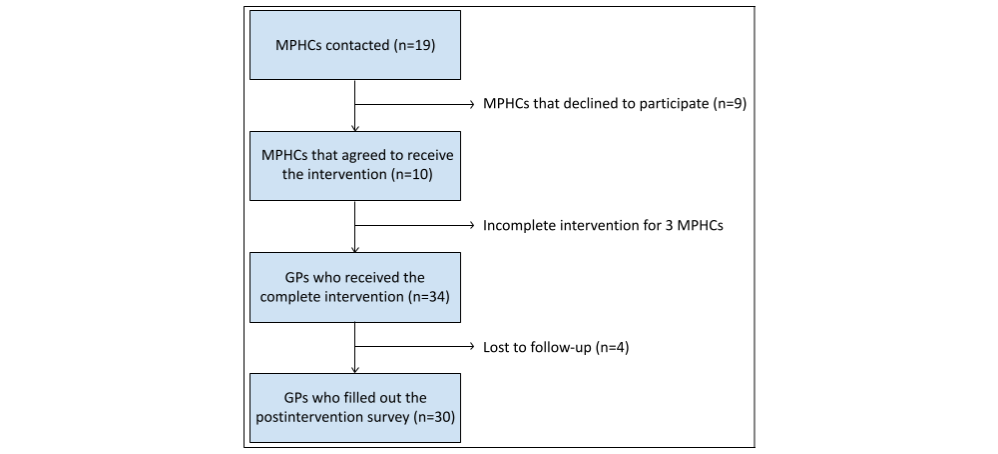
Participant flowchart. GP: general practitioner; MPHC: multiprofessional health center.

The flow of participants is detailed in Figure 2.

### Baseline Data

Centers that did not respond were comparable to those that did respond in size and location (urban vs rural). However, responding centers appeared to be more familiar with thesis work, including those involving university internship supervisors. Among the 10 centers that agreed to participate, 5 (50%) included university internship supervisors, compared with 1 among the 9 centers that declined.

The characteristics of the participants are summarized in Table 1.

**Table 1:** Sociodemographic characteristics of participants (n=34).

Variables		Participants, n (%)
**Sex**		
	Male	17 (50)
	Female	17 (50)
	Intersex	0 (0)
**Age group (y)**		
	21-30	7 (20.6)
	31-40	14 (41.2)
	41-50	7 (20.6)
	51-60	3 (8.8)
	>60	3 (8.8)
**Professional status**		
	GP^a^	33 (97.1)
	GP resident	1 (2.9)
**Experience (y)**		
	0-10	21 (61.8)
	11-20	7 (20.6)
	21-30	3 (8.8)
	≥31	3 (8.8)
**Practice location**		
	Urban	24 (71)
	Semirural	10 (29.4)
	Rural	0 (0)
**Pulmonologist in the MPHC^b^**		
	Yes	11 (32.4)
	No	20 (58.8)
	Do not know	3 (8.8)
**Previous ecoresponsible training**		
	Yes	6 (17.6)
	No	26 (76.5)
	Do not know	2 (5.9)
**Willing to change prescribing habits**		
	Strongly agree	19 (55.9)
	Agree	11 (32.4)
	Somewhat agree	2 (5.9)
	Do not know	2 (5.9)
	Not at all in agreement	0 (0)

^a^GP: general practitioner.

^b^MPHC: multiprofessional health center.

Participants who did not complete the study were mostly male (3/4, 75%) and older than 50 years, in contrast to the study population, which was balanced by sex (17/34, 50% male and 17/34, 50% female) with a median age of 30 to 40 years.

The complete-case sensitivity analysis produced results comparable to the primary analysis, with largely overlapping CIs, suggesting that missing follow-up data did not materially influence the results.

### Primary Outcome

A total of 34 participants completed all 6 clinical cases, resulting in a total of 204 responses. The proportion of responses including a pMDI significantly decreased from 37.7% (77/204) at baseline to 4.4% (9/204) after the intervention (*P*<.001; Table 2).

Thirty-four participants completed the 2 questions on reliever prescription, resulting in a total of 68 responses. Reliever responses including a pMDI decreased from 70.6% (48/68) to 4.4% (3/68) (*P*<.001), representing a marked reduction.

Thirty-four participants completed the 4 questions on maintenance prescription, resulting in a total of 136 responses. Maintenance treatment responses including a pMDI decreased from 21.3% (29/136) to 4.4% (6/136; *P*=.003), representing a substantial reduction.

We found no evidence of heterogeneity in the effect of the intervention across the predefined subgroups (age, sex, years of practice, practice location, and presence of a pulmonologist within the health care center). No statistically significant interaction was observed for any of these variables, suggesting that the effect of the intervention on the primary outcome was consistent across subgroups. These analyses were considered exploratory and should be interpreted with caution.

**Table 2:** Primary and secondary outcome measures.

Outcome				Pretest, n (%; 95% CI)		Posttest, n (%; 95% CI)		*P* value
**pMDI^a^ responses**								
	Total prescribing responses^b^ (n=204)			77 (37.7; 31.0-44.7)		9 (4.4; 1.6-7.2)		<.001
	Reliever treatment^c^ (n=68)			48 (70.5; 60-81)		3 (4.4; 0-9.3)		<.001
	Maintenance treatment^d^ (n=136)			29 (21.3; 14.4-28.2)		6 (4.4; 1.0-7.9)		.003
**GINA^e^ adherence—asthma**								
	Reliever treatment (n=34)			2 (5.9; 0-13.8)		13 (38.2; 22-54)		.001
	Maintenance treatment (n=68)			24 (35.3; 24-47)		38 (55.9; 44-68)		.02
**GOLD^f^ adherence—chronic obstructive pulmonary disease**								
	Reliever treatment (n=34)			25 (73.5; 59-88)		31 (91.2; 82-100)		.06
	Maintenance treatment (n=68)			29 (42.6; 31-54)		32 (47.1; 35-59)		.61
	Inhaled corticosteroid			25 (36.8; 25-48)		20 (29.4; 19-40)		.79

^a^pMDI: pressurized metered-dose inhaler.

^b^Total prescribing responses included 6 questions per participant: 3 related to asthma and 3 related to chronic obstructive pulmonary disease, including 2 maintenance treatment questions and 1 reliever treatment question. This resulted in 204 prescribing responses.

^c^Reliever treatment included 2 questions: 1 for asthma and 1 for chronic obstructive pulmonary disease.

^d^Maintenance treatment included 4 questions: 2 for asthma and 2 for chronic obstructive pulmonary disease.

^e^GINA: Global Initiative for Asthma.

^f^GOLD: Global Initiative for Chronic Obstructive Lung Disease.

### Secondary Outcomes

Adherence to GINA asthma guidelines improved significantly, particularly regarding reliever therapy. Responses including inhaled corticosteroid-formoterol increased from 5.9% (2/34) to 38.2% (13/34; *P*=.001), with a corresponding decrease in responses, including SABA.

The analysis of adherence to GOLD guidelines for COPD is detailed in Table 2.

The response grid can be found in Multimedia Appendix 4.

At baseline, GPs highlighted 3 main determinants for inhaler choice: treatment efficacy (25/34, 73.5%), ease of use (23/34, 67.6%), and patient characteristics such as age (18/34, 52.9%).

These 3 determinants remained stable after the educational intervention. However, the proportion of participants reporting environmental impact as an influencing factor increased substantially from 2.9% (1/34) to 52.9% (18/34).

No variable was significantly associated with the magnitude of change in intended prescribing responses in post hoc analysis.

The proportion of participants who correctly identified the most polluting inhaler (pMDI) increased by 30 percentage points from 61.8% (21/34) to 91.2% (31/34; *P*=.001).

Satisfaction with the educational intervention was high: 91.2% (31/34) of participants reported being “very satisfied” with the training.

## Discussion

### Principal Results

Our study showed that the educational intervention improved participants’ knowledge regarding high-carbon-impact inhalers. Indeed, participants’ responses in the clinical vignettes included fewer high-carbon-impact inhalers following the intervention. These changes were observed across multiple prescribing scenarios, including asthma and COPD, as well as reliever and maintenance treatment questions.

This reduction was independent of participants’ sociodemographic characteristics, suggesting that the educational intervention had consistent exploratory effects across a diverse range of practitioners. Baseline prescribing responses were broadly aligned with national prescribing patterns [20], thereby supporting the external relevance of the findings.

The most pronounced changes were observed in reliever treatment scenarios. This is notable because SABA-based reliever strategies remain widely used in clinical practice. In asthma scenarios, the reduction in responses including SABA was accompanied by an increase in responses including inhaled corticosteroid-formoterol, rising from 5.9% (2/34) to 38.2% (13/34). This is consistent with current GINA recommendations aiming to reduce SABA overuse [19,34].

These findings support the feasibility of brief educational interventions aimed at improving awareness of environmentally responsible inhaler prescribing and adherence to asthma guidelines.

### Interpretation and Implications

The observed changes in questionnaire responses suggest that brief, structured educational interventions may improve GPs’ awareness of lower-carbon inhaler alternatives. Responses in asthma scenarios shifted away from SABA-based reliever strategies, consistent with current international guidelines [34].

However, no significant improvement was observed in COPD management. COPD management remains challenging in primary care practice. A 2015 report from the French national health authority estimated that only 10% to 30% of patients with COPD are diagnosed in France [13].

Several responses suggested potential confusion between asthma and COPD management strategies, particularly regarding the use of inhaled corticosteroids. These findings suggest that future educational interventions may benefit from a dedicated module specifically focused on COPD management.

To our knowledge, this is the first French study assessing GPs’ knowledge regarding the environmental impact of inhalers on the climate. Despite the modest sample size, participant satisfaction was high. These findings are consistent with data from the United Kingdom, where a quality improvement initiative reported a relative reduction of approximately 30% in pMDI SABA prescribing rates [24]. This may reflect growing interest in environmentally responsible prescribing among practitioners in coordinated care settings.

The findings also support the pedagogical value of small-group, structured educational sessions [35].

The intervention was evaluated using the Kirkpatrick model, particularly focusing on satisfaction and knowledge acquisition [36]. Participants reported high satisfaction and demonstrated measurable knowledge gains.

From a broader perspective, environmentally responsible inhaler prescribing aligns with current environmental and health care sustainability goals. Reducing pMDI use contributes to lower greenhouse gas emissions, complementing European Union regulations on hydrofluorocarbons [37]. Emerging technologies, such as HFA-152a propellants with substantially lower global warming potential, may further support this transition [38,39].

Further studies using real-world prescribing data are needed to determine whether these educational effects translate into sustained clinical practice changes.

### Limitations

Several limitations should be acknowledged. The educational assessment relied on only 2 theoretical clinical vignettes and therefore explored a limited range of prescribing situations encountered in primary care practice. Outcomes were self-reported and based on clinical vignettes rather than actual prescribing behavior, introducing potential social desirability and recall bias [40]. Objective prescription data, based on real-world data (eg, pharmacy claims), could provide more robust evidence of behavioral change and actual prescribing practices. Therefore, the findings should not be interpreted as evidence regarding the safety, efficacy, or real-world implementation of reducing pMDI prescribing. This study evaluated a brief educational intervention and was not designed to contribute to the literature regarding the safety or efficacy of changing inhaler prescribing strategies.

Indeed, the reduction in pMDI responses from 70% to 4% appears too large to reflect a true change in prescribing behavior; rather, it likely reflects improved knowledge of lower-carbon inhaler alternatives among participants. These results may be influenced by social desirability bias, the Hawthorne effect, and test-retest bias and should therefore be interpreted cautiously.

Additionally, the updated version of the Kirkpatrick model emphasizes the importance of catalysts—such as job aids, supervision, or incentives—for sustaining behavioral change [36]. These elements were not systematically integrated into our educational intervention but should be considered in future iterations.

The study population was younger than the national average for French GPs, likely reflecting the recruitment strategy through MPHCs, settings more frequently adopted by early-career physicians [41,42]. This may limit the generalizability of our findings. Moreover, the study population was relatively small and consisted of self-selected participants, which may have introduced a selection bias. Participating centers appeared to be more familiar with research and thesis-related activities, including hosting university-supervised trainees. This may have increased their willingness to engage in an educational intervention focused on environmentally sustainable prescribing practices.

Finally, the study used a pre-post design without a control group. In addition, prescribing responses across clinical scenarios were not fully independent, as multiple responses were provided by the same participants. While appropriate for exploratory and feasibility research, randomized controlled studies are needed to better assess the effectiveness of educational interventions aimed at promoting environmentally sustainable prescribing practices.

Patient and public involvement was not included in this study. As the research specifically targeted physicians’ practices, patient and public involvement was not initially considered. However, we acknowledge that involving patients or public contributors may have enriched the interpretation and broader relevance of the findings, and this will be explored in future research.

### Conclusions

To our knowledge, this is the first French pilot study evaluating an educational intervention focused on environmentally responsible inhaler prescribing in primary care.

Even if reducing pMDI prescriptions could lower CO_2_ emissions, the safety of switching a stable patient from a pMDI to a DPI for maintenance therapy remains widely debated. Although previous studies have shown overall safety in retrospective analyses [20], some recent publications have raised concerns regarding such switches in older patients [21]. Therefore, the transition between inhalers should be carried out in an individualized manner, based on evidence-based medicine principles while ensuring treatment safety and effectiveness for each patient.

These findings suggest that a brief educational session may improve GPs’ awareness of lower-carbon inhaler alternatives and adherence to asthma guidelines in theoretical prescribing scenarios. This may represent a first step toward decreasing pMDI prescriptions while simultaneously improving adherence to asthma management recommendations.

Future implementation strategies could build on the growing network of MPHCs and Territorial Primary Care Communities (Communautés Professionnelles Territoriales de Santé) in France [43-45], promoting sustainable health care practices.

Future work should explore broader implementation, integration of technological and systemic enablers, and long-term sustainability of behavior change, ensuring safe, effective, and ecoresponsible prescribing.

## Data Availability

The datasets generated and analyzed during this study are not publicly available but are available from the corresponding author on reasonable request. Interested researchers may contact the corresponding author by email to obtain access.

## Appendix

Multimedia Appendix 1 CONSORT checklist for pilot and feasibility studies.

Multimedia Appendix 2 Presentation slides with commentary.

Multimedia Appendix 3 Preintervention and postintervention questionnaire.

Multimedia Appendix 4 Questionnaire response grid.

## Abbreviations

CONSORT: Consolidated Standards of Reporting Trials
COPD: chronic obstructive pulmonary disease
DPI: dry powder inhaler
GINA: Global Initiative for Asthma
GOLD: Global Initiative for Chronic Obstructive Lung Disease
GP: general practitioner
HFA: hydrofluoroalkane
MPHC: multiprofessional health center
pMDI: pressurized metered-dose inhaler
SABA: short-acting beta-2 agonist
SMI: soft mist inhaler
