# Supplementary material for: Educational Intervention on Environmentally Responsible Inhaler Prescribing Among French General Practitioners: Pilot Pre-Post Study
Source: JMIR Form Res. 2026 Jun 11;10:e89593. doi: 10.2196/89593 (PMC13305471; doi:10.2196/89593)
Supplement: Multimedia Appendix 2 [file formative_v10i1e89593_app2.pdf]

Département
de Médecine
Générale
Faculté de Médecine
Montpellier-Nîmes

UNIVERSITÉ MONTPELLIER

How to prescripe inhalers to reduce greenhouse gas emission in treatement of asthma and COPD ?

By Lapeyre Camille General medicine intern at the university of Montpellier
Under the direction of Dr François Carbonnel

Part 1 : Global warming :

Part 2 : Inhalers :

Part 3 : In practice :

Part 1 : Global Warming :

1- Greenhouse gas (GHG) :

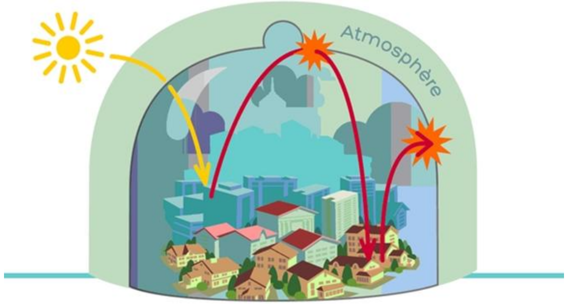

2- Global Warming Potential (GWP) :

- **Medical GHS :**
  - **HFA 134a : GWP 100 = 1 300 eq-CO2**
  - **HFA 227ea : GWP 100 = 3 350 eq-CO2**
  - **HFA 152a : GWP 100 = 138 eq-CO2**

GHG	GWP for 100 years
CO_2	1
CH_4	23
N_2O	296
HFC - 23	12 000
HFC – 134a	1 300
SF_6	22 200

Source: *IPCC Third Assessment Report* (2001).

CO2 emissions in the healthcare sector in France

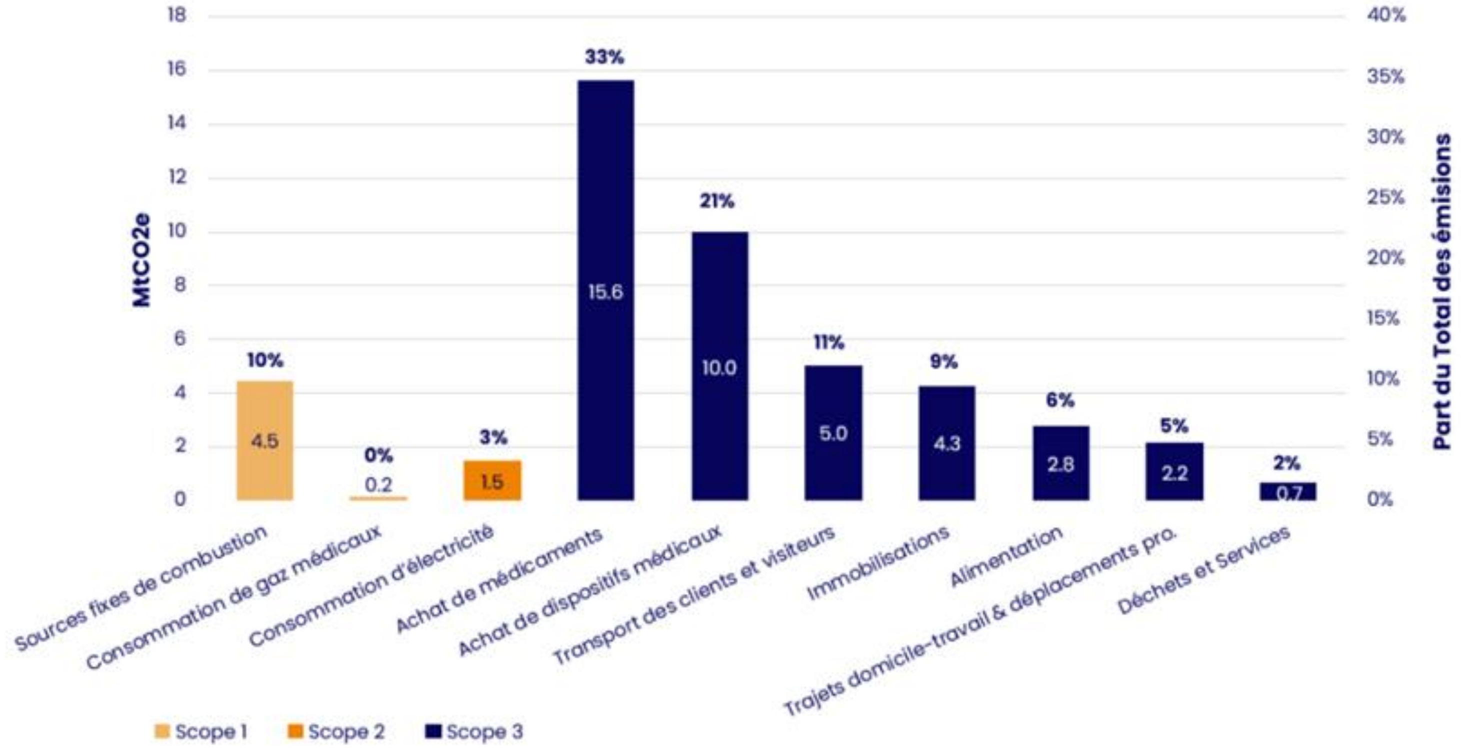

Figure 27 - Répartition des émissions du secteur de la santé (MtCO2e)

Source : The Shift Project

Limit warming to +2°C ?

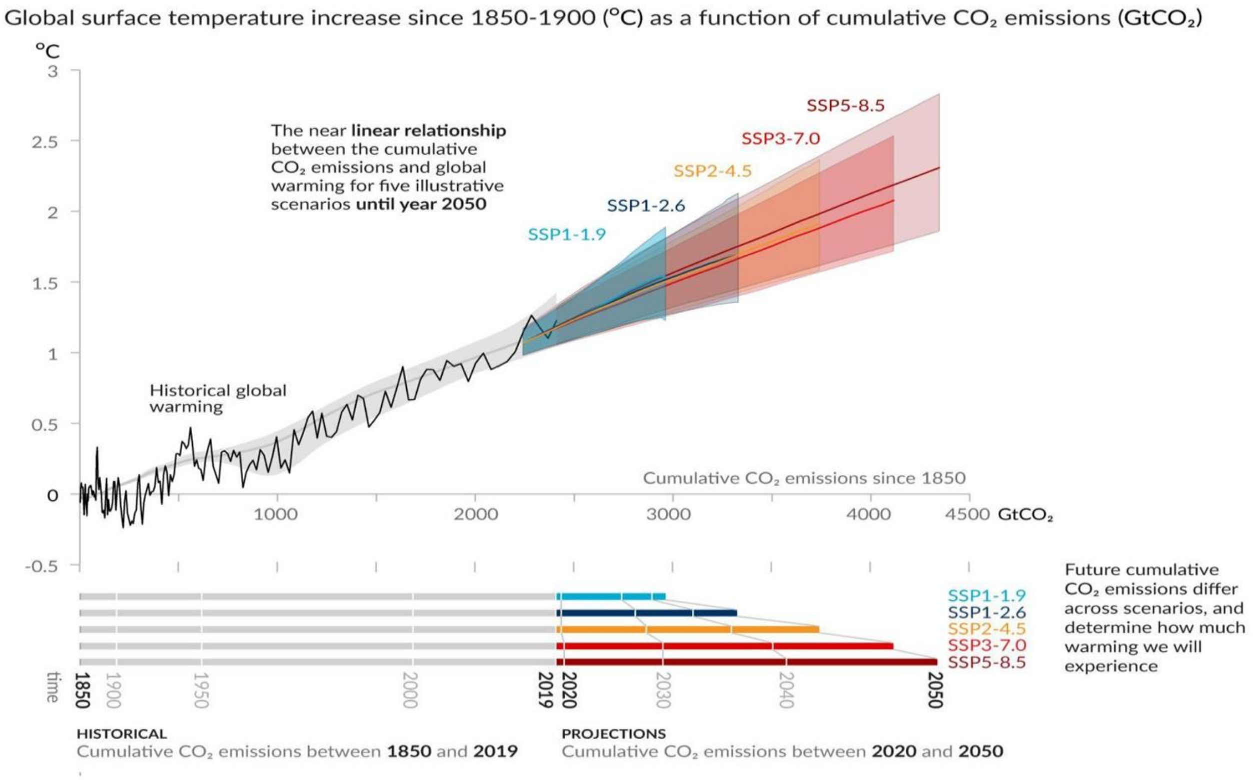

- **2020 : 2 500 GtCO2 emitted, namely 85% of permitted emissionsby 2050 (and after…)**

Consequences of global warming :

- GIEC 2020 : With warming of +2°C, current heat waves will be 14 times more frequent, tropical corals will have disappeared, as will half of the French Forest.

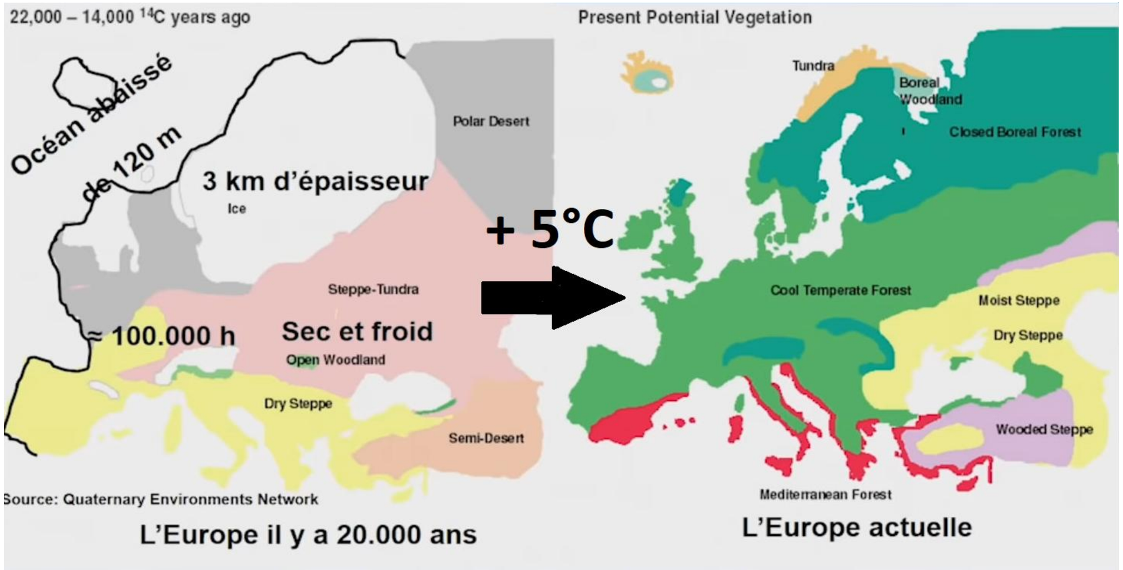

- In 5 000 years increase of 5°C = + 1°C per millennium = birth of agriculture and sedentarization of humans.
- Currently : increase of + 1 to 5°C per century !

Part 2 : Inhalers :

pressured Metered-dose Inhaler<br><br>Or « pMDI »	Dry Powder Inhaler<br><br>Or « DPI »	Soft Mist Inhaler<br><br>Or « SMI »
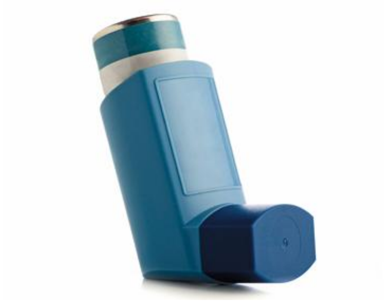	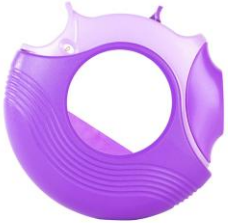 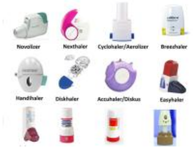	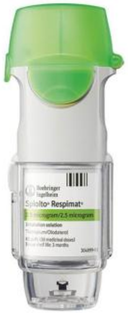

Carbon footprint of inhalers :

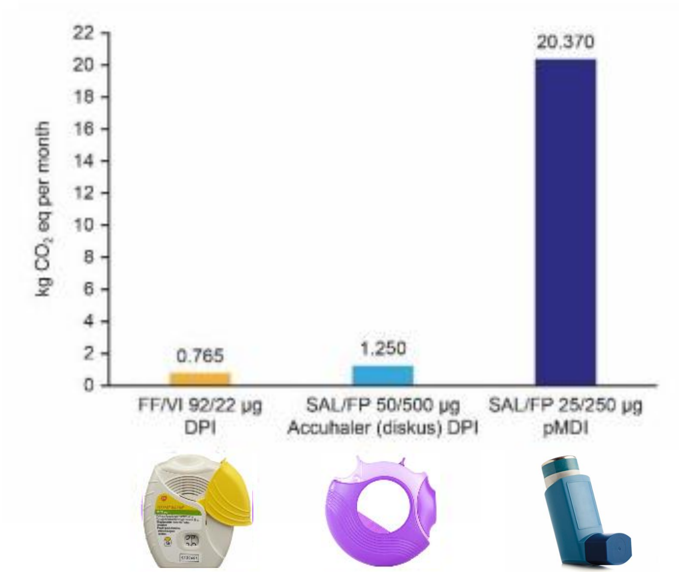

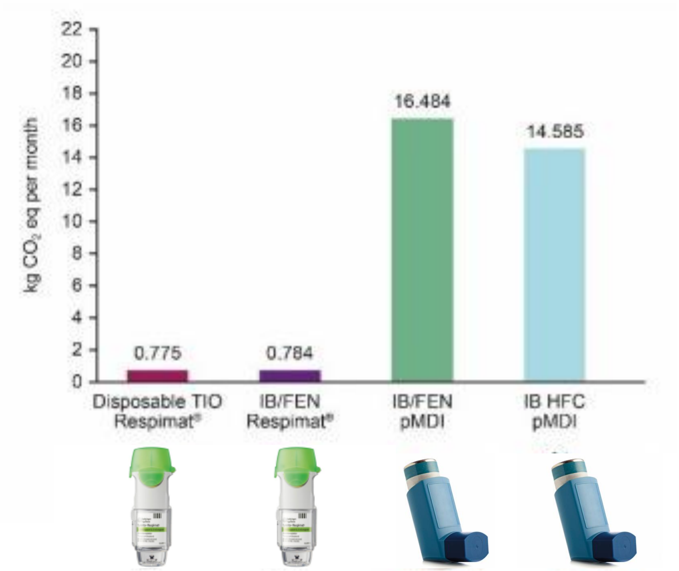

Source : Woodcock A, Beeh KM, Sagara H, Aumônier S, Addo-Yobo E, Khan J, et al. The environmental impact of inhaled therapy: making informed treatment choices. Eur Respir J. 16 déc 2021.

- **« pMDI » have a GHG effect between 15 and 30 times greater than « DPI » or « SMI »**

What CO2 savings per patient/per year ? :

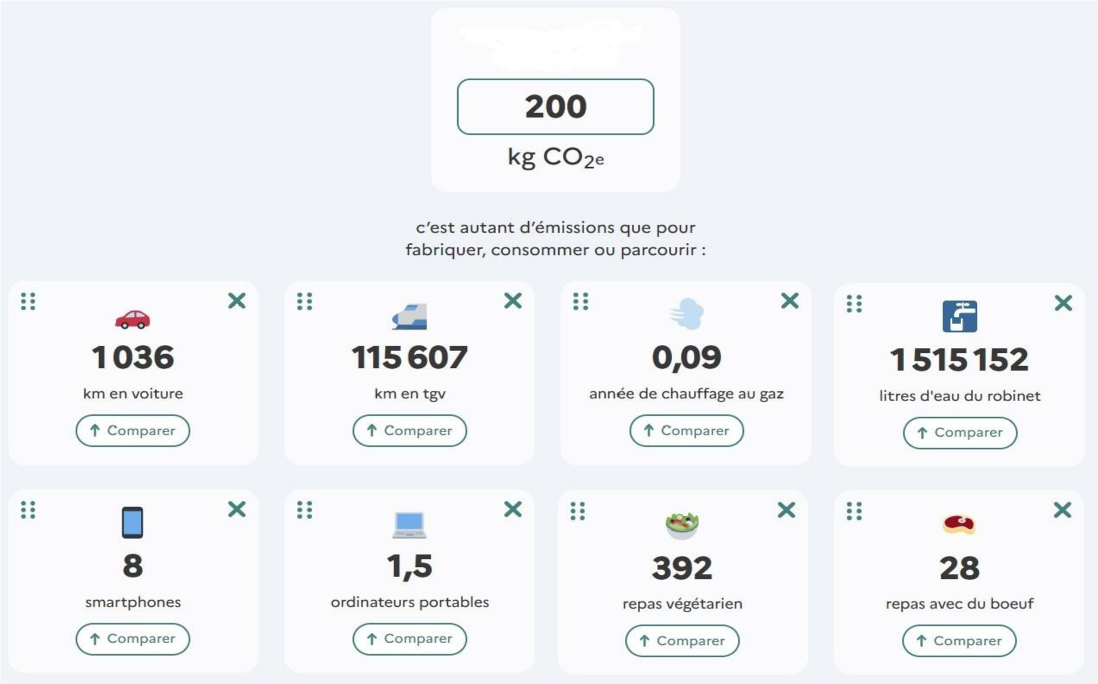

Source : Pernigotti D, Stonham C, Panigone S, Sandri F, Ferri R, Unal Y, et al. Reducing carbon footprint of inhalers: analysis of climate and clinical implications of different scenarios in five European countries. BMJ Open Respir Res. déc 2021;8(1):e001071.

- **200 kg CO_2 = 1 year of vegetarian meal = 1 month of meal with beef**
- **200 kg CO_2 = 1 way Montpellier-Paris by car = 100 trips Montpellier-Paris by train**

Quelles économies de CO2 par patient/par an ? :

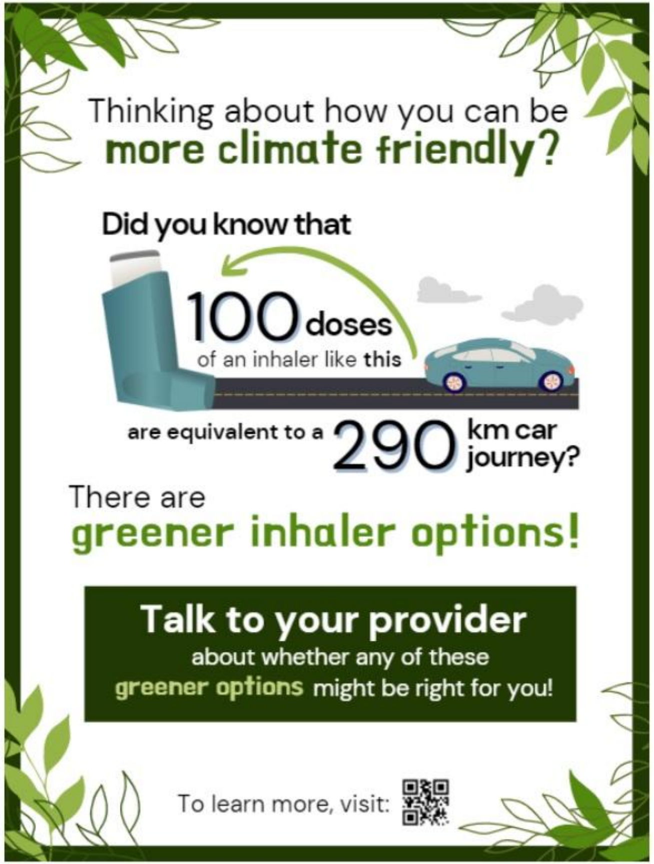

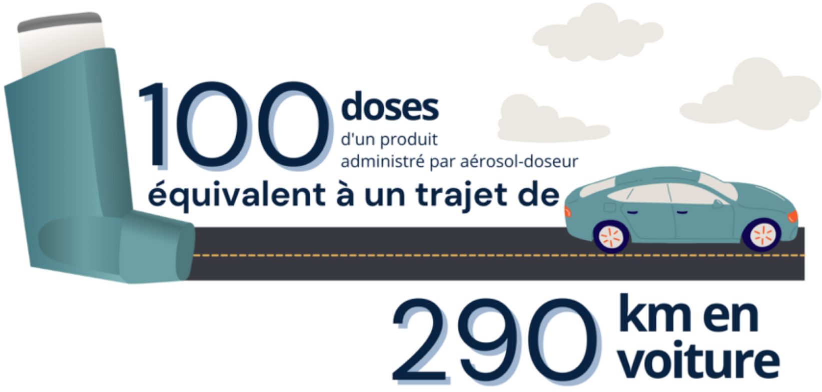

Clinical effectiveness of devices :

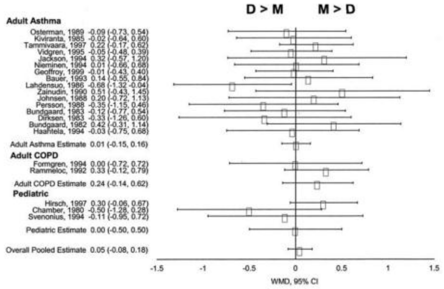

FIGURE 7. Weighted standardized mean difference for combined end point (FEV_1, PEFR, or sGaw) in outpatient β_2-agonist trials comparing MDI (M) vs DPI (D). See the legend of Figure 2 for abbreviations not used in the text.

“DPI” versus “pMDI” :

Maintenance treatement:

- **2005 Méta-analyses of 25 articles : No significant difference between “DPI” and “pMDI”**

Dolovich MB, Ahrens RC, Hess DR, Anderson P, Dhand R, Rau JL, et al. Device selection and outcomes of aerosol therapy: Evidence-based guidelines: American College of Chest Physicians/American College of Asthma, Allergy, and Immunology. Chest. janv 2005;127(1):335-71.

Reliever treatement:

- **2014 Review of 25 articles : “DPI” just as effective as “pMDI” et nébulization**

Selroos O. Dry-powder inhalers in acute asthma. Therapeutic Delivery. janv 2014;5(1):69-81.

“pMDI” versus “SMI” :

- **2004 Review of 5 articles : “SMI” is not inferior to “pMDI” in COPD**

Dr Frank Kässner, Rick Hodder, Eric D. Bateman (2004). *A Review of Ipratropium Bromide/Fenoterol Hydrobromide (Berodual®)Delivered Via Respimat® Soft Mist™ Inhaler in Patients with Asthma and Chronic Obstructive Pulmonary Disease*

Which inhaler for which patient ?

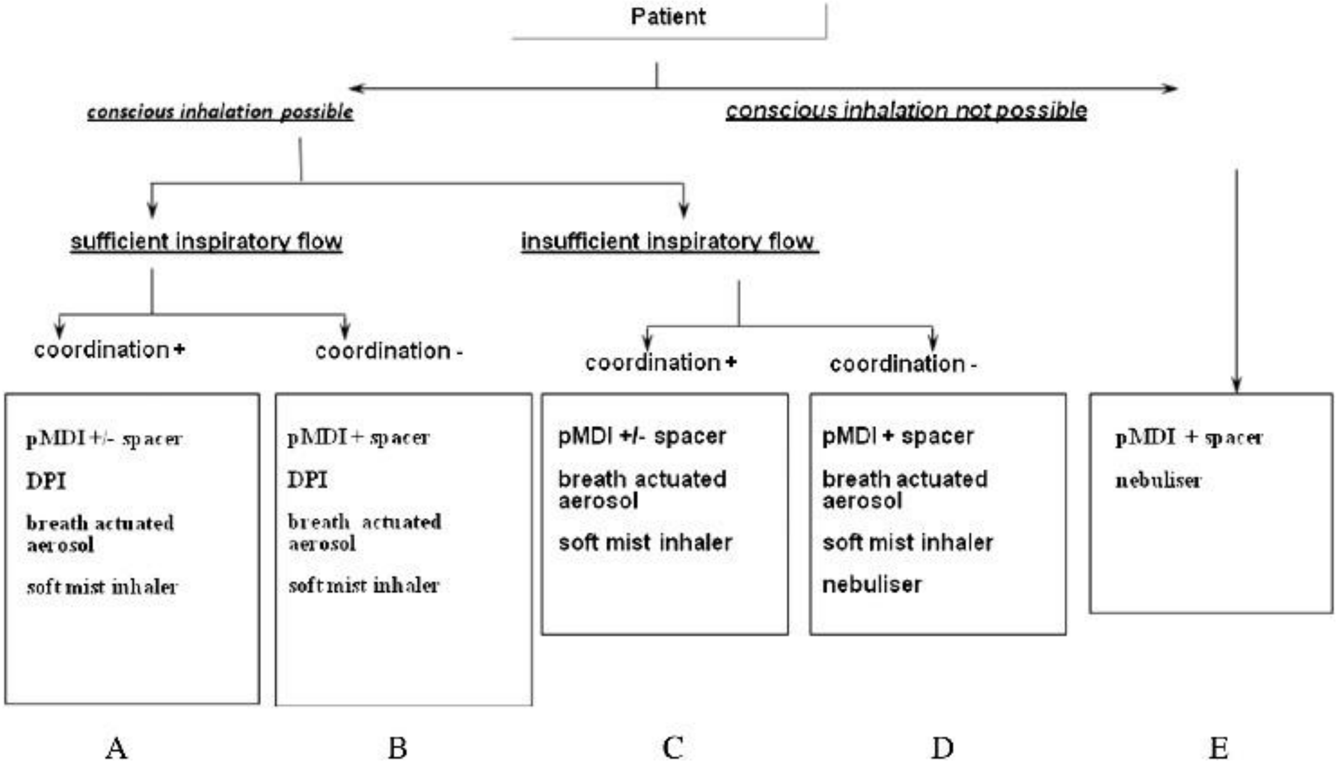

- **“DPI” not usable in children < 6 years old and elderly patient (inspiratory flow <30L/min)**
- **“pMDI” not usable in children < 8 years old et coordination issue**

Inhaler prescription :

In Europe :

- **SABA : 60% are “pMDI”**
- **Sweden 10% are “pMDI” vs UK 70% are “pMDI”**

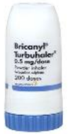

Bricanyl® Turbuhaler® (Terbutaline)

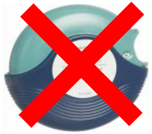

Ventoline® Diskus® (Salbutamol)

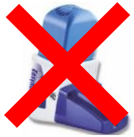

Buventol® Easyhaler® (Salbutamol)

In Ile de France (Paris) :

- **Maintenance treatement : 74% “DPI” + “SMI”**
- **Reliever treatement : 94% “pMDI”**

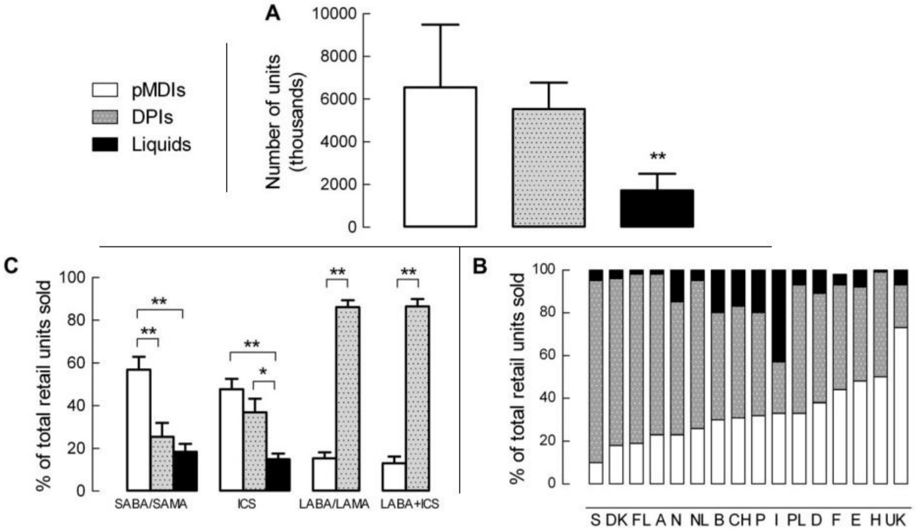

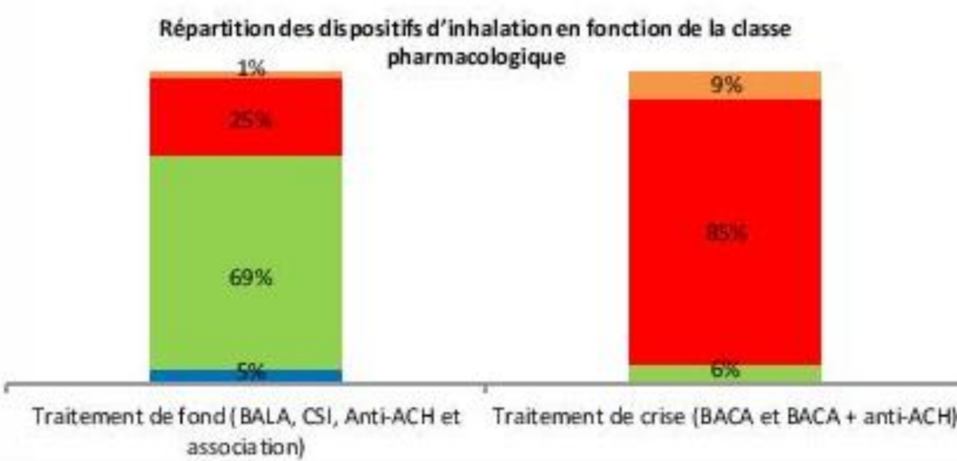

How to reduce the carbon footprint of inhaler ?

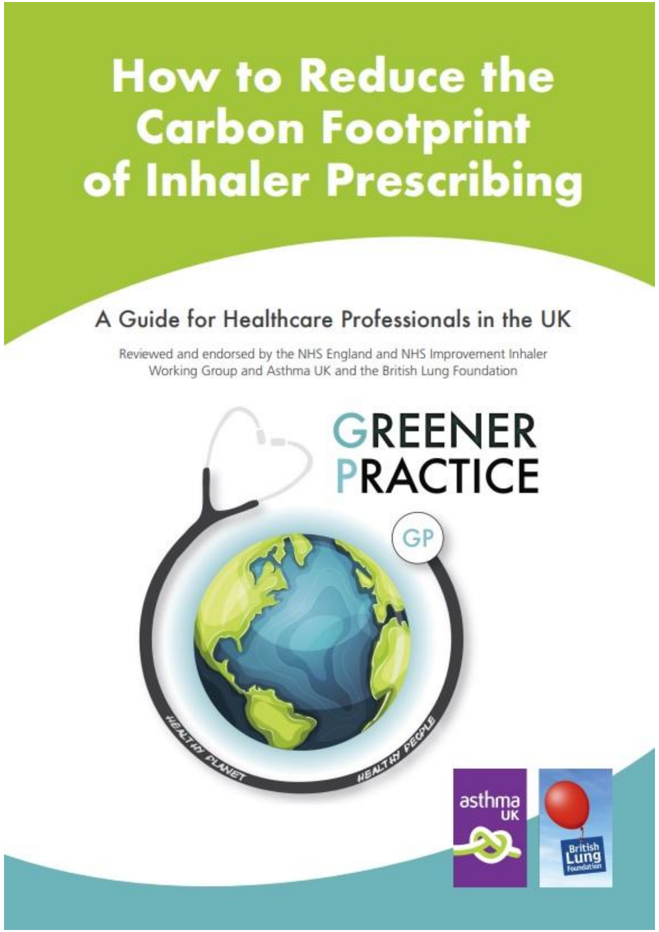

1) <u>Optimize the treatment if ACT < 20:</u>
- Review the technique of device usage
- Change the background treatment

<u>2) Use a DPI or SMI as a first choice, unless :</u>
- The patient is unable to take a deep and fast breath: Children < 6 years old, elderly individuals.
- The patient prefers using a pMDI
- The patient is well-controlled with a pMDI
- If exacerbations are frequent and there is concern that the patient may not be able to use a DPI. In this case, prescribe a pMDI with a spacer.

<u>3) Prescribe the minimum number of puffs:</u>
- For example: 1 puff of INNOVAIR 200ug corresponds to 2 puffs of INNOVAIR 100ug.

Thorax 2022 : A, Janson C, Rees J, Frith L, Löfdahl M, Moore A, et al. Effects of switching from a metered dose inhaler to a dry powder inhaler on climate emissions and asthma control: post-hoc analysis. Thorax 7 févr 2022

- **Out of 1080 patients, 916 kept the prescribed DPI.**

Greenhouse Gas Emission Reductions in Europe: Various Scenarios :

- Reducing aerosol-dose inhaler emissions in the United Kingdom, Germany, France, Spain, Italy. (**SABA/SAMA exclued**)

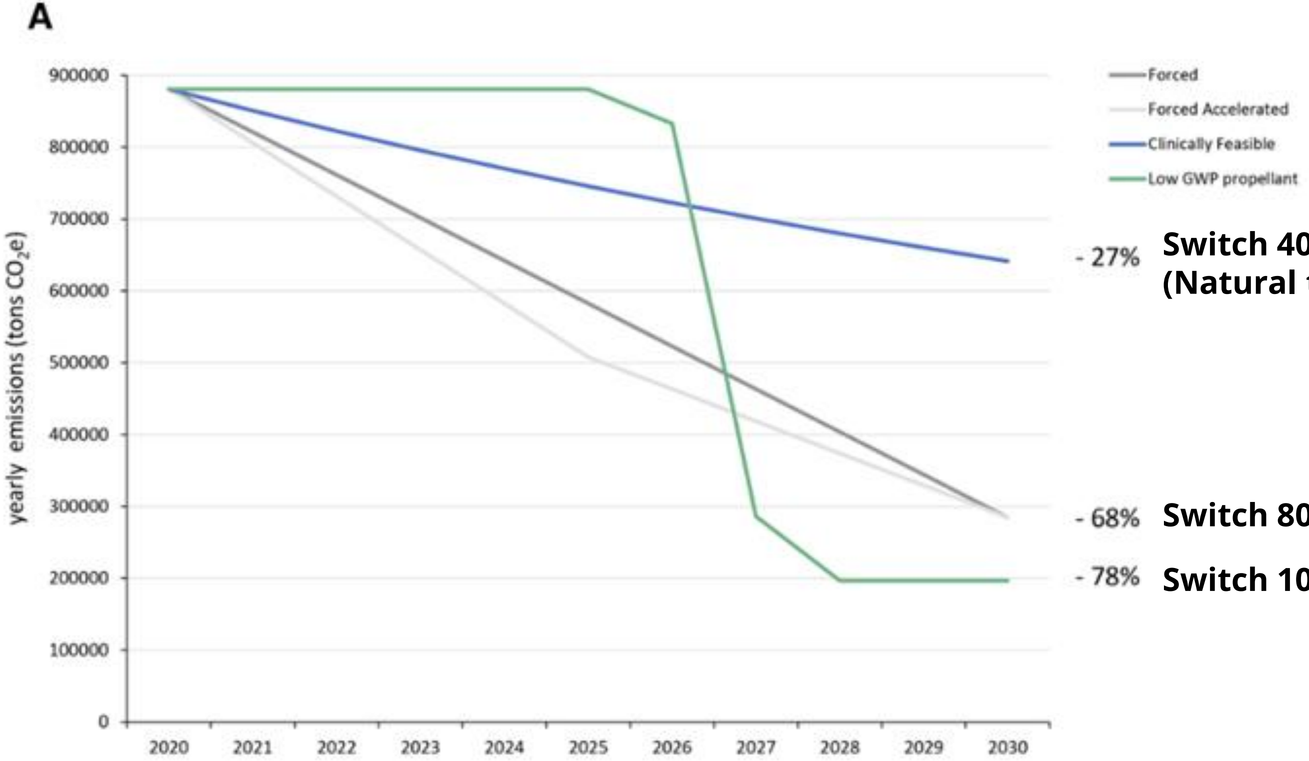

Switch 40% DPI/SMI (Natural trend)

Switch 80% DPI/SMI

Switch 100% HFA 152a

- **Reduction of 600 tons/CO2/year = 20,000 smartphones.**

Greenhouse Gas Emission Reductions in Europe: Various Scenarios :

- Reducinf the overuse of SABA :

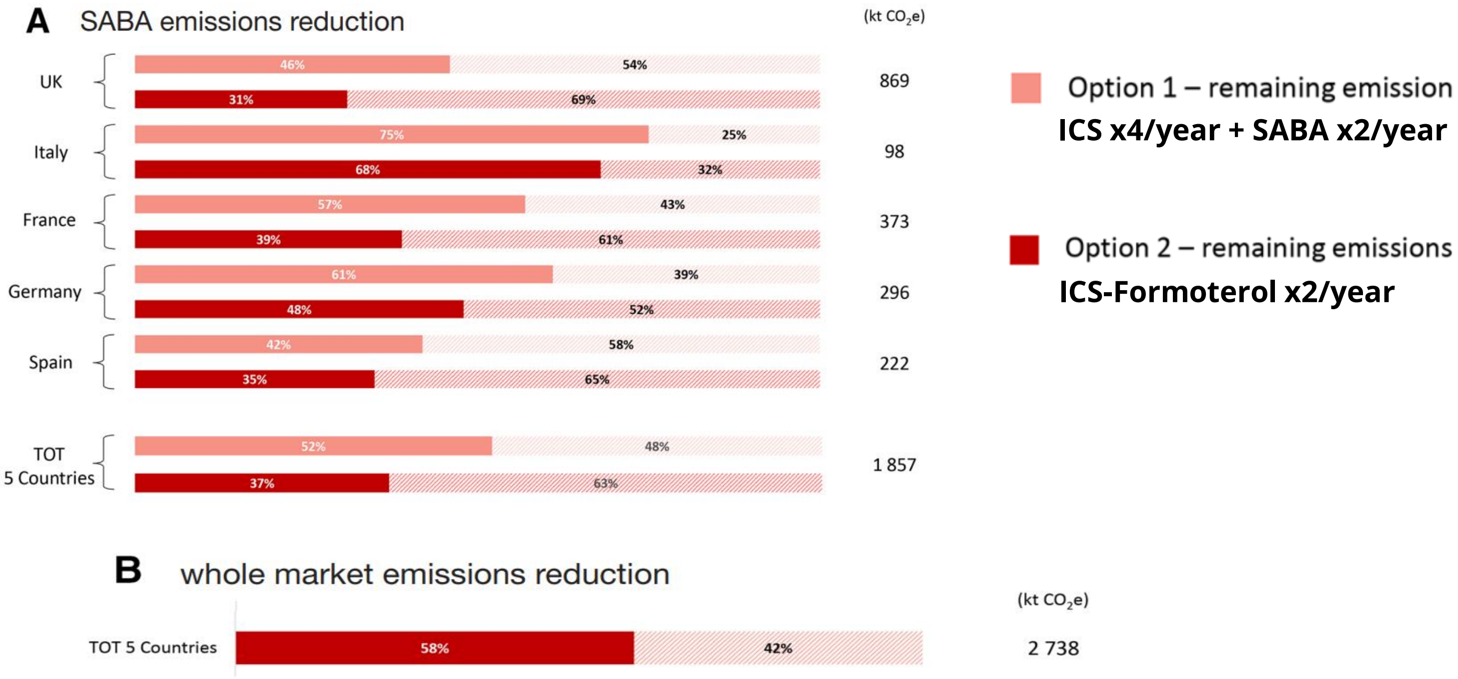

- **Reduction of 42% in emissions, equivalent to 2,738 tons/CO2/year = 86,000 smartphones.**

Part 3 : In pratice :

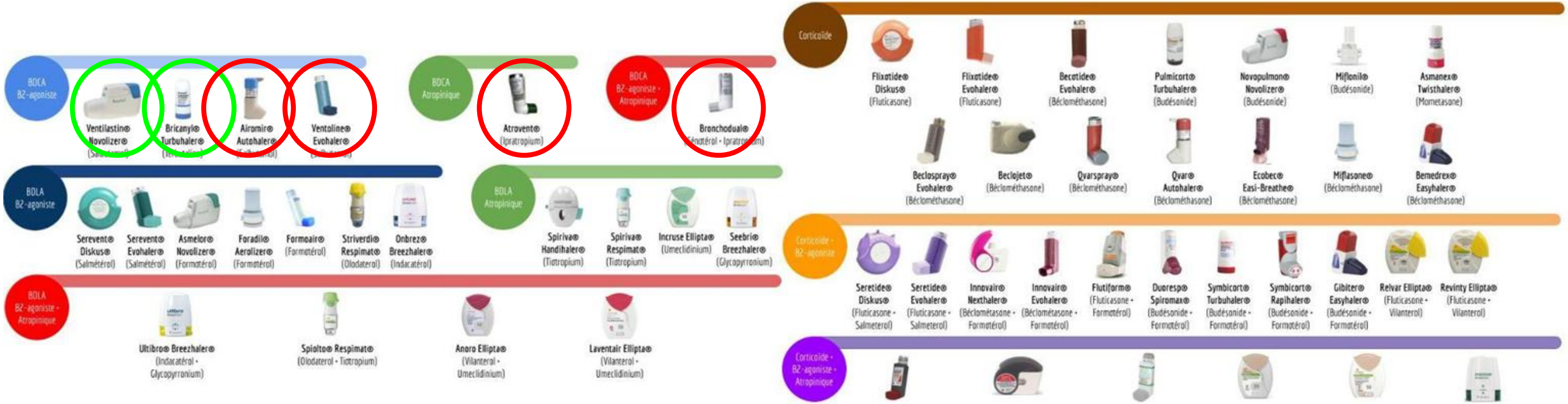

- **In total, there are 54 marketed specialties in France (including only 6 SABA/SAMA)**

Clinical case 1 :

You receive a young asthmatic patient in their twenties for a consultation. They are not on any maintenance treatment, but he mentions using a short-acting bronchodilator like Salbutamol three to four times a week when they feel breathless. Last month, he woke up twice in the middle of the night with wheezing cough episodes.

1/ What first-line maintenance treatment do you prescribe?

=> Step 2 : low-dose ICS :

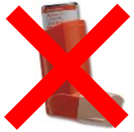

Flixotide®
Evohaler®

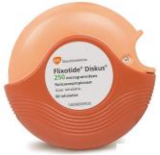

Flixotide®
Diskus®
(Fluticasone)

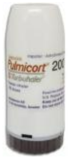

Pulmicort®
Turbuhaler®
(Budésonide)

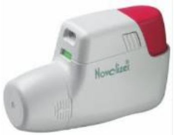

Novopulmon®
Novolizer®
(Budésonide)

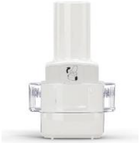

Miflonil®
(Budésonide)

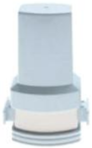

Miflasone®
(Béclométhasone)

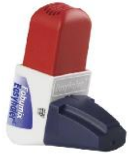

Bemedrex®
Easyhaler®
(Béclométhasone)

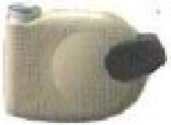

Beclojet®
(Béclométhasone)

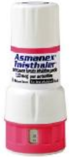

Asmanex®
Twisthaler®
(Mometasone)

=> Or low-dose ICS + Formoterol = AIR-only : 8 inhalation per day

Clinical case 1 :

2/ What reliever treatment do you prescribe?

=> SABA « as needed » :

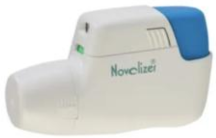

Ventilastin® Novolizer®
(Salbutamol)

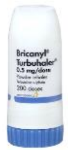

Bricanyl® Turbuhaler®
(Terbutaline)

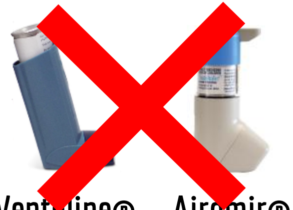

Ventoline® Evohaler®

Airomir® Autohaler®

=> Or low-dose ICS + Formoterol = AIR-only : 8 inhalation per day

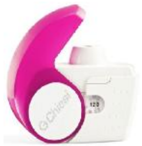

Innovair® Nexthaler®
(Béclométasone + Formotérol)

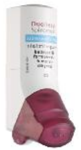

Duoresp® Spiromax®
(Budésonide + Formotérol)

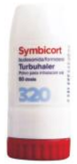

Symbicort® Turbuhaler®
(Budésonide + Formotérol)

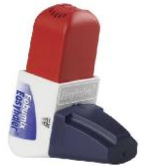

Gibiter® Easyhaler®
(Budésonide + Formotérol)

Clinical case 1 :

The patient returns 3 months later. Despite your treatment, which includes inhaled corticosteroids prescribed at an optimal dose, he continues to feel uncomfortable. An escalation of the treatment seems justified to you. As a second-line option, what maintenance treatment do you introduce ?

=> Step 3 : low-dose ICS + LABA :

=> Ou low-dose ICS + Formoterol= MART

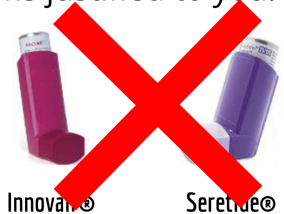

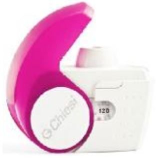

Innovair® Nexthaler® (Béclométasone + Formotérol)

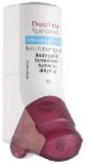

Duoresp® Spiromax® (Budésonide + Formotérol)

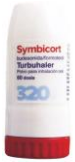

Symbicort® Turbuhaler® (Budésonide + Formotérol)

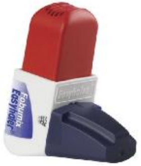

Gibiter® Easyhaler® (Budésonide + Formotérol)

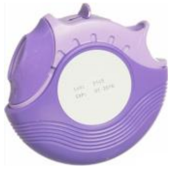

Seretide® Diskus® (Fluticasone + Salmeterol)

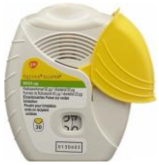

Relvar Ellipta® (Fluticasone + Vilanterol)

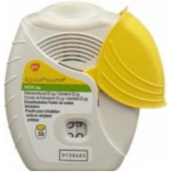

Revinty Ellipta® (Fluticasone + Vilanterol)

Recommendation for Asthma Management:

GINA 2023 :

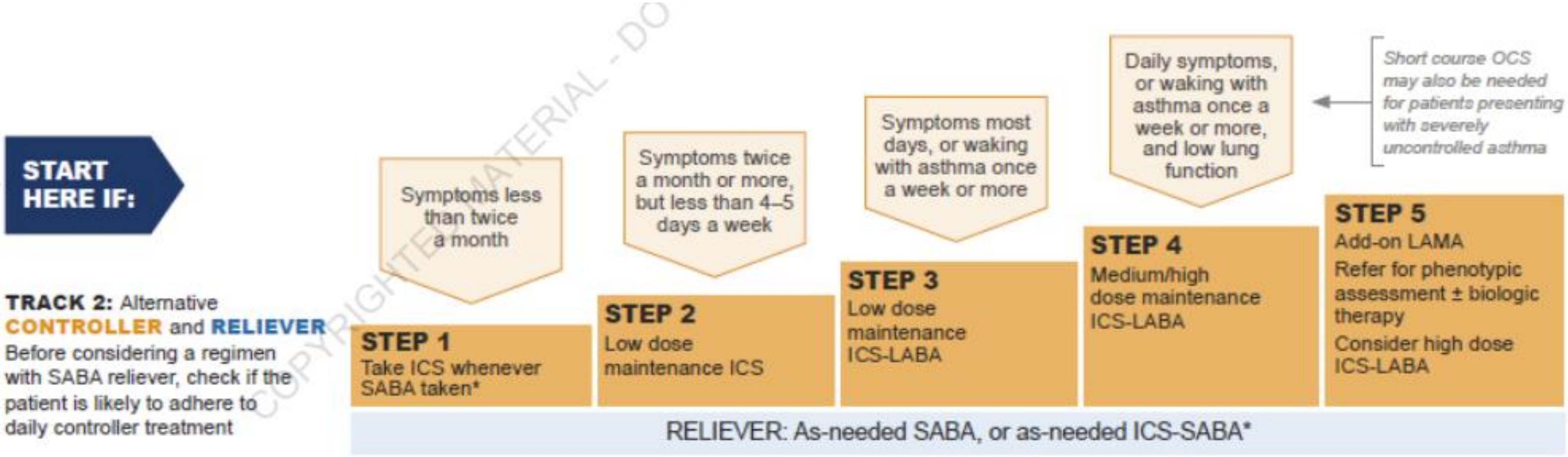

GINA 2023	AIR-only (ICS-Formotérol)	MART (ICS-Formotérol)

GINA 2023 Recommendations (from age 6) :

Clinical Benefits:

Step 1 to 2 : AIR-only (Anti-Inflammatory Reliever) :

ICS-Formoterol « **as-needed** » before exercise or exposure to allergens

- **Reduction of 65% in exacerb/hospitalizations compared to SABA alone and 37% compared to ICS alone.**

Step 3 à 5 : MART (Maintenance And Reliever Therapy) :

ICS-Formoterol as **maintenance** and **as needed**

- **Reduction of 20% in exacerbations/hospitalizations**

Ecological Benefits:

- **Reduction of 42% in CO_2 emissions**

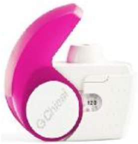

Innovair®
Nexthaler®

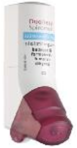

Duoresp®
Spiromax®

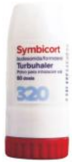

Symbicort®
Turbuhaler®

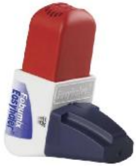

Gibiter®
Easyhaler®

Follow-up of an asthmatic patient:

1. In the past **4 weeks**, how much of the time did your **asthma** keep you from getting as much done at work, school or at home?

All of the time (1)	Most of the time (2)	Some of the time (3)	A little of the time (4)	None of the time (5)

2. During the past **4 weeks**, how often have you had shortness of breath?

More than once a day (1)	Once a day (2)	3 to 6 times a week (3)	Once or twice a week (4)	Not at all (5)

3. During the past **4 weeks**, how often did your **asthma** symptoms (wheezing, coughing, shortness of breath, chest tightness or pain) wake you up at night or earlier than usual in the morning?

4 or more nights a week (1)	2 or 3 nights a week (2)	Once a week (3)	Once or twice (4)	Not at all (5)

4. During the past **4 weeks**, how often have you used your rescue inhaler or nebulizer medication (such as albuterol)?

3 or more times per day (1)	1 or 2 times per day (2)	2 or 3 times per week (3)	Once a week or less (4)	Not at all (5)

5. How would you rate your **asthma** control during the **past 4 weeks**?

Not controlled at all (1)	Poorly controlled (2)	Somewhat controlled (3)	Well controlled (4)	Completely controlled (5)

SCORE

TOTAL

Copyright 2002, by QualityMetric Incorporated.
Asthma Control Test is a trademark of QualityMetric Incorporated.

- **Example : Treatement of asthma**

Step 1 :

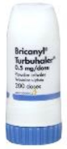

Bricanyl® Turbuhaler® (Terbutaline)

Step 2 :

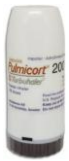

Pulmicort® Turbuhaler® (Budésonide)

Step 3 :

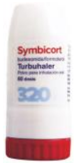

Symbicort® Turbuhaler® (Budésonide + Formotérol)

Clinical case 2 :

You receive a fifty-year-old patient who is a smoker. He explains that a pulmonologist diagnosed him with COPD several months ago and advised him to consult his general practitioner if he experienced shortness of breath. For several weeks now, he has been feeling breathless when walking with friends of his age, and sometimes he must stop to catch his breath. He does not describe any recent exacerbation episodes.

4/ What first-line maintenance treatment do you prescribe?

=> <u>LABA (B2 agoniste) :</u>

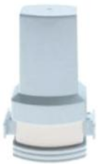

Foradil®
Aerolizer®
(Formotérol)

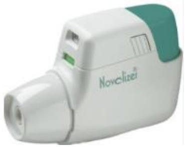

Asmelor®
Novolizer®
(Formotérol)

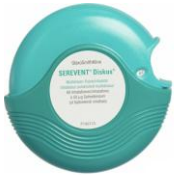

Serevent®
Diskus®
(Salmétérol)

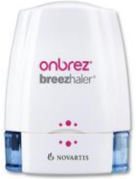

Onbrez®
Breezhaler®
(Indacatérol)

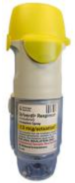

Striverdi®
Respimat®
(Olodaterol)

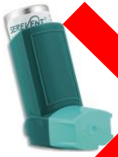

Serevent®
Evohaler®

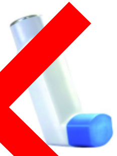

Formoair®
Evohaler®

Clinical case 2 :

5/ What reliever treatment do you prescribe?

=> <u>SABA « as needed »:</u>

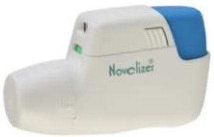

Ventilastin®
Novolizer®
(Salbutamol)

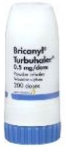

Bricanyl®
Turbuhaler®
(Terbutaline)

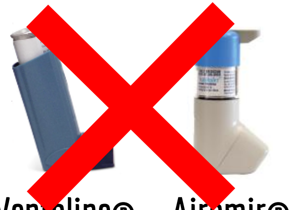

Ventoline®
Evohaler®

Airomir®
Autohaler®

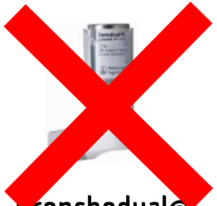

Bronchodual®
Evohaler®

Clinical case 2 :

The patient returns 3 months later. Despite your treatment, he still complains of breathlessness. He still does not describe any exacerbation episodes.

6/What second-line maintenance treatment do you prescribe?
=> Combination of LABA + LAMA (B2 agonist + Anticholinergic) :

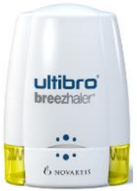

Ultibro® Breezhaler®
(Indacatérol +
Glycopyrronium)

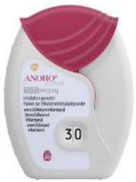

Anoro Ellipta®
(Vilanterol +
Umeclidinium)

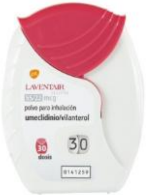

Laventair Ellipta®
(Vilanterol +
Umeclidinium)

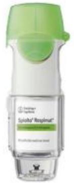

Spiolto® Respimat®
(Olodaterol +
Tiotropium)

Recommendations for COPD management:

Recommandation GOLD 2023:

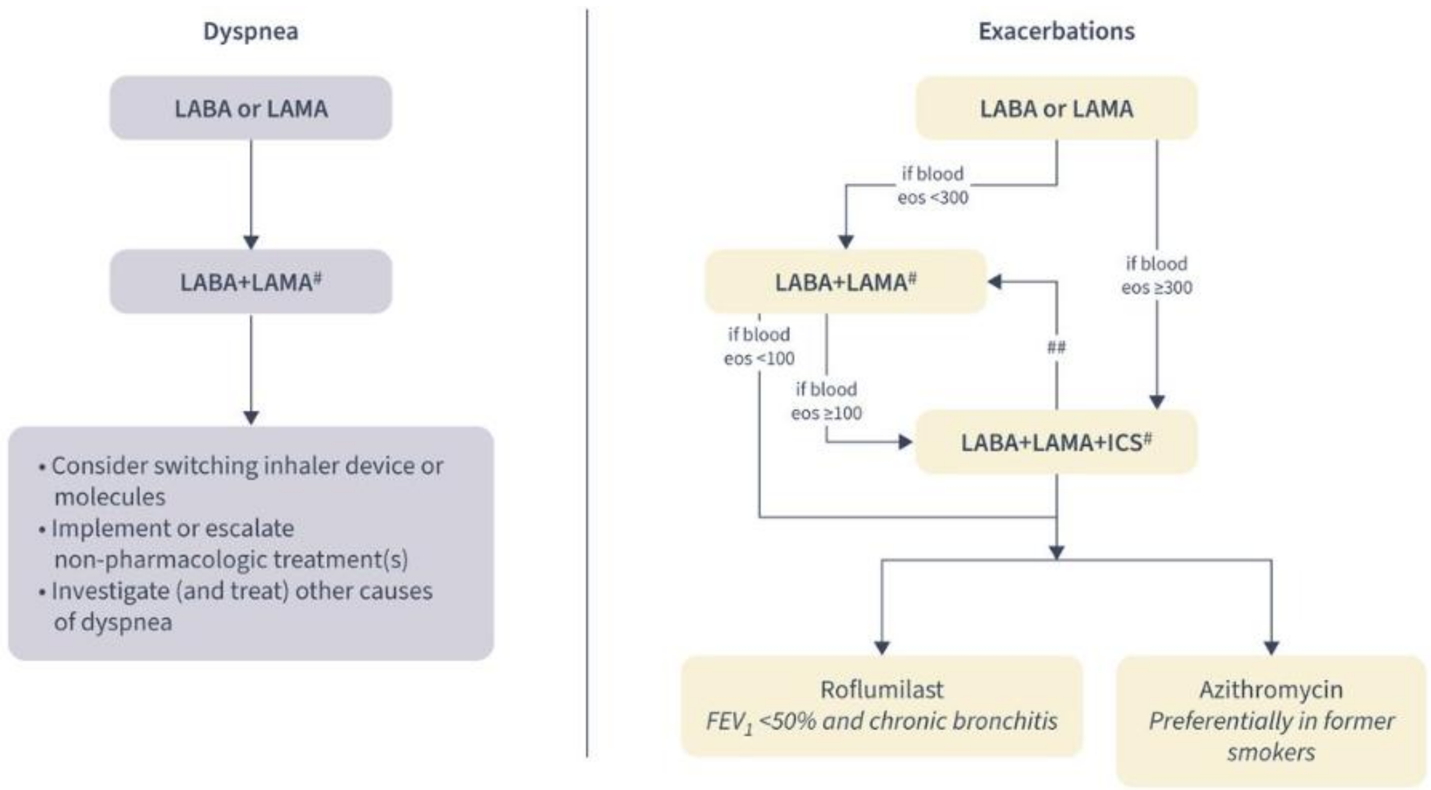

COPD treatement:

- **Example : Treatement of COPD**

SABA	LABA (B2-ago)	LAMA (Anticho)	LABA+LAMA	LABA + ICS

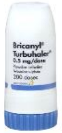

Bricanyl® Turbuhaler®

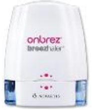

Onbrez® Breezhaler®

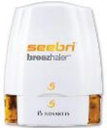

Seebri® Breezhaler®

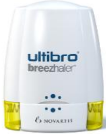

Ultibro® Breezhaler®

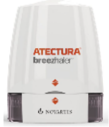

Atectura® Breezhaler®

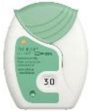

Incruse® Ellipta®

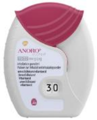

Anoro® Ellipta®

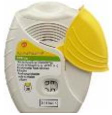

Relvar® Ellipta®

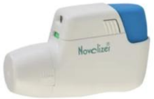

Ventilastin® Novolizer®

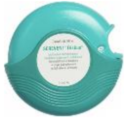

Serevent® Diskus®

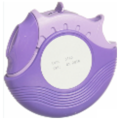

Seretide® Diskus®

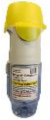

Striverdi® Respimat®

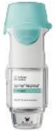

Spiriva® Respimat®

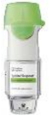

Spiolto® Respimat®

Key Messages :

- **Reliever treatement : 95% of “pMDI”**

→ **“DPI”**

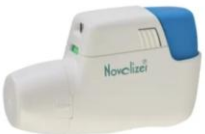

Ventilastin®
Novolizer®

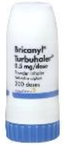

Bricanyl®
Turbuhaler®

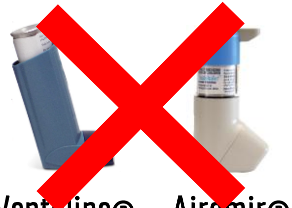

Ventoline®
Evohaler®

Airomir®
Autohaler®

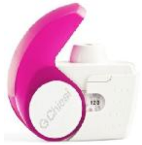

Innovair®
Nexthaler®

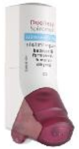

Duoresp®
Spiromax®

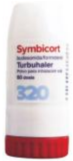

Symbicort®
Turbuhaler®

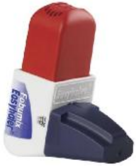

Gibiter®
Easyhaler®

- **Treatement instead of « pMDI”**

→ **“SMI”**

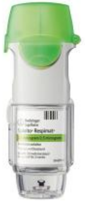

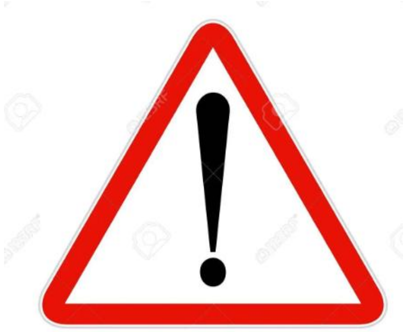

Questionnaire POST –test in 3 months

VIDEO PRESENTATION

- https://youtu.be/uR8yJ_QGJbc?si=xIehmQfmi9CsEaJO
