# Supplementary material for: Educational Intervention on Environmentally Responsible Inhaler Prescribing Among French General Practitioners: Pilot Pre-Post Study
Source: JMIR Form Res. 2026 Jun 11;10:e89593. doi: 10.2196/89593 (PMC13305471; doi:10.2196/89593)
Supplement: Multimedia Appendix 3 [file formative_v10i1e89593_app3.pdf]

QUESTIONNIARE PRE-Test 1

Hello, my name is Camille LAPEYRE, and I am a general medicine intern at the Montpellier Faculty. Thank you for taking the time to participate.
This anonymized questionnaire is part of my thesis research, conducted in the Occitanie Est region, under the guidance of Dr. François CARBONNEL, Associate Professor of General Medicine at the University.
The objective of this questionnaire is to gather information about your practices and knowledge regarding inhalation devices used in asthma and COPD before participating in a brief 20-minute intervention on their environmental impact. A second questionnaire will be emailed to you in 3 months to assess the impact of this intervention.

This questionnaire consists of 19 questions and two short clinical cases. It should not take more than 10 minutes of your time.

Participants Characteristics:
1 / Please write your first and last name:

…………………………………………………………………………………
2/ What is your gender?
Please select only one of the following options:
- Female - Male - Other

3/In which group of age do you belong?
Please select only one of the following options:
- 20-30 years - 31-40 years - 41- 50 years - 51-60 years - 61 years and more

4/ For how long have you been practicing?
Please select only one of the following options:
- Less than 10 years - 10-20 years - 21-30 years - 31 – 40 years - 41 years and more

5/ What is your type of professional practice?
Please select only one of the following options:
- Urban - Rural - Semi-Rural - Other

6/ What is your professional status?
Please select only one of the following options:
- Physician - Medical Intern - Nurse - Other

Knowledge about inhalation devices:
7/ Do you think inhalation devices can have a negative impact on the environement?
Please select only one of the following options:
- Yes - Non - Don’t know

8/ In your opinion, which inhalation devices do you consider the most polluting?
Please select only one of the following options:
- Dry powder inhaler (example: Ellipta®, Bricanyl®…)
- Pressured metered inhaler (example: Ventoline®, Airomir®…)

- Soft Mist Inhaler (example: Respimat®…)
- Don’t know

9/ When prescribing a inhalation device, what criteria influence your choice?
Choose 3 criteria from the list:
- Therapeutic efficiency
- Characteristics of the patient (age, respiratory capacity …)
- Ease of use
- Patient’s choice
- Prescription habit
- Environmental impact

Clinical case 1:
You receive a young asthmatic patient in their twenties for a consultation. They are not on any maintenance treatment, but he mentions using a short-acting bronchodilator like Salbutamol three to four times a week when they feel breathless. Last month, he woke up twice in the middle of the night with wheezing cough episodes.

10/ What first-line maintenance treatment do you prescribe? (Please write the commercial name of the device and, if possible, the type of inhaler):

………………………………………….
11/ What "as-needed" treatment do you prescribe? (Please write the commercial name of the device and, if possible, the type of inhaler):

………………………………………….
12/ The patient returns 3 months later. Despite your treatment, which includes inhaled corticosteroids prescribed at an optimal dose, he continues to feel uncomfortable. An escalation of the treatment seems justified to you. As a second-line option, what maintenance treatment do you introduce? (Write the commercial name of the device and, if possible, the type of inhaler):
…………………………………………..

Clinical case 2:
You receive a fifty-year-old patient who is a smoker. He explains that a pulmonologist diagnosed him with COPD several months ago and advised him to consult his general practitioner if he experienced shortness of breath. For several weeks now, he has been feeling breathless when walking with friends of his age, and sometimes he must stop to catch his breath. He does not describe any recent exacerbation episodes.

13/ What first-line maintenance treatment do you prescribe? (Please write the commercial name of the device and, if possible, the type of inhaler):

………………………………………….
14/ What "as-needed" treatment do you prescribe? (Please write the commercial name of the device and, if possible, the type of inhaler):

……………………………………….
15/ The patient returns 3 months later. Despite your treatment, he still complains of breathlessness. He still does not describe any exacerbation episodes. What second-line maintenance treatment do you prescribe? (Write the commercial name of the device and, if possible, the type of inhaler):

……………………………………….

Environmental sensitivity characteristics:

16/ Is a pneumologist part of the MSP (multidisciplinary health center)?
Please select only one of the following options:

- Yes - No - Don’t know

17/ Do you have care protocols or public health initiatives related to chronic respiratory diseases within your MSP?
Please select only one of the following options:

- Yes - No - Don’t know

18/ Have you ever received training or awareness on eco-responsibility in general medical practice?
Please select only one of the following options:

- Yes - No - Don’t know

19/ Are you willing to change your prescription habits to reduce their environmental impact?
Please select only one of the following options:

- Strongly disagreed - Somewhat disagreed - Agree – Completely agreed - Don’t know

QUESTIONNAIRE POST-test 2

Hello, my name is Camille LAPEYRE, and I am a general medicine intern at the Montpellier Faculty. Thank you for taking the time to participate.
This anonymized questionnaire is part of my thesis research, conducted in the Occitanie Est region, under the guidance of Dr. François CARBONNEL, Associate Professor of General Medicine at the University.
The objective of this questionnaire is to gather information about your practices and knowledge regarding inhalation devices used in asthma and COPD before participating in a brief 20-minute intervention on their environmental impact. A second questionnaire will be emailed to you in 3 months to assess the impact of this intervention.

This questionnaire consists of 15 questions and two short clinical cases. It should not take more than 10 minutes of your time.

Participants Characteristics:
1 / Please write your first and last name:

………………………………………………………………………………

Clinical case 1:
You receive a young asthmatic patient in their twenties for a consultation. They are not on any maintenance treatment, but he mentions using a short-acting bronchodilator like Salbutamol three to four times a week when they feel breathless. Last month, he woke up twice in the middle of the night with wheezing cough episodes.

2/ What first-line maintenance treatment do you prescribe? (Please write the commercial name of the device and, if possible, the type of inhaler):

…………………………………………
3/ What "as-needed" treatment do you prescribe? (Please write the commercial name of the device and, if possible, the type of inhaler):

…………………………………………
4/ The patient returns 3 months later. Despite your treatment, which includes inhaled corticosteroids prescribed at an optimal dose, he continues to feel uncomfortable. An escalation of the treatment seems justified to you. As a second-line option, what maintenance treatment do you introduce? (Write the commercial name of the device and, if possible, the type of inhaler):
…………………………………………

Clinical case 2:
You receive a fifty-year-old patient who is a smoker. He explains that a pulmonologist diagnosed him with COPD several months ago and advised him to consult his general practitioner if he experienced shortness of breath. For several weeks now, he has been feeling breathless when walking with friends of his age, and sometimes he must stop to catch his breath. He does not describe any recent exacerbation episodes.

5/ What first-line maintenance treatment do you prescribe? (Please write the commercial name of the device and, if possible, the type of inhaler):

……………………………………….
6/ What "as-needed" treatment do you prescribe? (Please write the commercial name of the device and, if possible, the type of inhaler):

…………………………………….
7/ The patient returns 3 months later. Despite your treatment, he still complains of breathlessness. He still does not describe any exacerbation episodes. What second-line maintenance treatment do you prescribe? (Write the commercial name of the device and, if possible, the type of inhaler):

…………………………………….

Impact of the training :
8/ Do you think inhalation devices can have a negative impact on the environement?
Please select only one of the following options:
- Yes - Non - Don't know

9/ In your opinion, which inhalation devices do you consider the most polluting?
Please select only one of the following options:
- Dry powder inhaler (example: Ellipta®, Bricanyl®…)
- Pressured metered inhaler (example: Ventoline®, Airomir®…)
- Soft Mist Inhaler (example: Respimat®…)
- Don't know

10/ When prescribing a inhalation device, what criteria influence your choice?
Choose 3 criteria from the list:
- Therapeutic efficiency
- Characteristics of the patient (age, respiratory capacity …)
- Ease of use
- Patient's choice
- Prescription habit
- Environmental impact

11/ Have you received any other training on the management of respiratory diseases in the last three months?
Please select only one of the following options:
- Yes - Non - Don't know

12/ Have you changed your prescription habits?
Please select only one of the following options:
- Not at all - A little - Moderately - Significantly

13/ Were you satisfied with the presentation provided?
Please select only one of the following options:
- Not at all - A little - Moderately - Significantly

14/ Have you already used the materials distributed during the presentation (asthma and COPD treatment recommendations)?
Please select only one of the following options:
- Not at all - A little - Moderately - Significantly

15/ You can write your comments

……………………………………………….
