# Supplementary material for: Educational Intervention on Environmentally Responsible Inhaler Prescribing Among French General Practitioners: Pilot Pre-Post Study
Source: JMIR Form Res. 2026 Jun 11;10:e89593. doi: 10.2196/89593 (PMC13305471; doi:10.2196/89593)
Supplement: Multimedia Appendix 4 [file formative_v10i1e89593_app4.pdf]

Appendix 3: Response Grid

<u>Clinical Case 1: Asthma</u>

"You are seeing an asthmatic patient in their twenties. They are not on any maintenance treatment, but they explain that they use a short-acting bronchodilator, such as Salbutamol, three to four times a week when they feel short of breath. Last month, they woke up twice in the middle of the night due to wheezing episodes.

Question 1: What maintenance treatment do you prescribe as a first-line option? (Please write the brand name of the device and, if possible, the type of inhaler).
Expected answers:

- GINA recommendations "ICS-formoterol."

Question 2: What reliever treatment do you prescribe?
Expected answers:

- GINA recommendations "ICS-formoterol."

Question 3: The patient returns three months later. Despite your treatment, which includes inhaled corticosteroids prescribed at an optimal dosage, they still feel short of breath. An increase in treatment seems justified. What second-line maintenance treatment do you introduce?
Expected answers:

- GINA recommendations "ICS-formoterol."

<u>Clinical Case 2: COPD</u>

"You are seeing a patient in consultation, a smoker in his fifties. He explains that a pulmonologist diagnosed him with COPD several months ago. The pulmonologist advised him to visit his general practitioner if he felt short of breath. For several weeks now, he has been feeling breathless when walking with friends of his age, and he sometimes must stop to catch his breath. He does not report any recent exacerbation episodes.

- **Question 1:** What first-line maintenance treatment would you prescribe?
    - **GOLD recommendations**: "Long-acting bronchodilator (LABA) of the B2 type or an anticholinergic."

Note: Dual therapies combining an inhaled corticosteroid (ICS) were excluded. Indeed, this treatment is not indicated in the absence of exacerbation signs and increases the risk of pneumonia by 50%.

- **Question 2:** What relieve treatment would you prescribe?
    - "Short-acting bronchodilator (SABA) of the B2 type or anticholinergic."

- **Question 3:** The patient returns 3 months later. Despite your treatment, he is still complaining of shortness of breath. He still does not report any exacerbation episodes. What second-line maintenance treatment would you prescribe?
    - Given the persistence of dyspnea despite monotherapy, an intensification of the maintenance treatment is expected:
    - **GOLD recommendations**: "Dual therapy combining two long-acting bronchodilators (LABA)."

Note: Dual therapies combining an ICS were excluded."
